# Successful surgical intervention for acute pyothorax caused by methicillin-resistant Staphylococcus aureus thoracic pyogenic spondylitis: a case report

**DOI:** 10.1186/s44215-024-00138-6

**Published:** 2024-02-22

**Authors:** Naoya Kitamura, Yoshifumi Shimada, Hayato Futakawa, Hiroto Makino, Yusuke Takegoshi, Hitoshi Kawasuji, Keitaro Tanabe, Toshihiro Ojima, Koichiro Shimoyama, Yoshihiro Yamamoto, Yoshiharu Kawaguchi, Tomoshi Tsuchiya

**Affiliations:** 1 Department of Thoracic Surgery, Toyama University Hospital, 2630, Sugitani, Toyama City, Toyama 930-0194 Japan; 2 Department of Orthopaedic Surgery, Faculty of Medicine, University of Toyama, 2630, Sugitani, Toyama City, Toyama 930-0194 Japan; 3 Department of Clinical Infectious Diseases, Graduate School of Medicine and Pharmaceutical Sciences, University of Toyama, 2630, Sugitani, Toyama City, Toyama 930-0194 Japan

**Keywords:** Acute pyothorax, Anterior thoracic fixation, Curettage of the intrapleural abscess, Methicillin-resistant staphylococcus aureus, Pyogenic spondylitis

## Abstract

**Background:**

Pyogenic spondylitis or intervertebral discitis rarely spreads into the thoracic cavity, resulting in pyothorax. Moreover, no study has reported methicillin-resistant *Staphylococcus aureus* (MRSA) as a cause. Conservative and surgical treatments are reportedly effective for the above-mentioned situations; however, there have been no comprehensive reports owing to the disease’s rarity. This report described a case of acute pyothorax due to MRSA-caused pyogenic spondylitis in which surgical intervention with curettage of the intrapleural abscess and simultaneous thoracic vertebral debridement and anterior fixation were effective.

**Case presentation:**

A 60-year-old female with Parkinson’s disease was diagnosed with pyogenic spondylitis caused by MRSA and managed with antibiotics. Subsequently, a right encapsulated pleural effusion was observed, and thoracentesis was performed. No bacteria were identified in the pleural fluid culture; nonetheless, the leukocytes in the fluid increased, and the patient was diagnosed with right acute pyothorax caused by pyogenic spondylitis. Management of the spondylitis and pyothorax before the disease became severe was necessary.

We performed curettage of the intrapleural abscess and vertebral debridement and anterior fixation using an autogenous rib through open thoracotomy. The inflammation or accompanying symptoms did not worsen 3 months after hospital discharge.

**Conclusions:**

Acute pyothorax is rare but may develop from pyogenic spondylitis, for which MRSA is a rarer causative agent. Simultaneous vertebral debridement and anterior fixation, with curettage of the thoracic cavity abscess, may be useful in its management.

## Background

Pyogenic spondylitis or intervertebral discitis rarely spreads into the thoracic cavity, leading to pyothorax [1–5]. *Staphylococcus aureus* [1], *Mycobacterium tuberculosis* [6–8], and *Streptococcus gordonii* [2] have been reported as causative agents. However, no study has reported methicillin-resistant *Staphylococcus aureus* (MRSA)-caused pyogenic spondylitis. Conservative and surgical treatments effectively treat acute pyothorax caused by pyogenic spondylitis [2–4, 7, 9, 10]; however, there are no comprehensive reports on this due to the disease’s rarity. Furthermore, various studies have reported surgical treatments, such as those in which only pyothorax curettage [2, 8, 11] or vertebral body fixation was performed [3]. However, few studies have reported these procedures being performed simultaneously. Therefore, no consensus has been made on the surgical strategy [2, 3, 5].

Herein, we report an acute pyothorax case caused by MRSA pyogenic spondylitis in which curettage of the abscess in the thoracic cavity and vertebral debridement and anterior fixation were effective.

## Case presentation

**Fig. 1:**
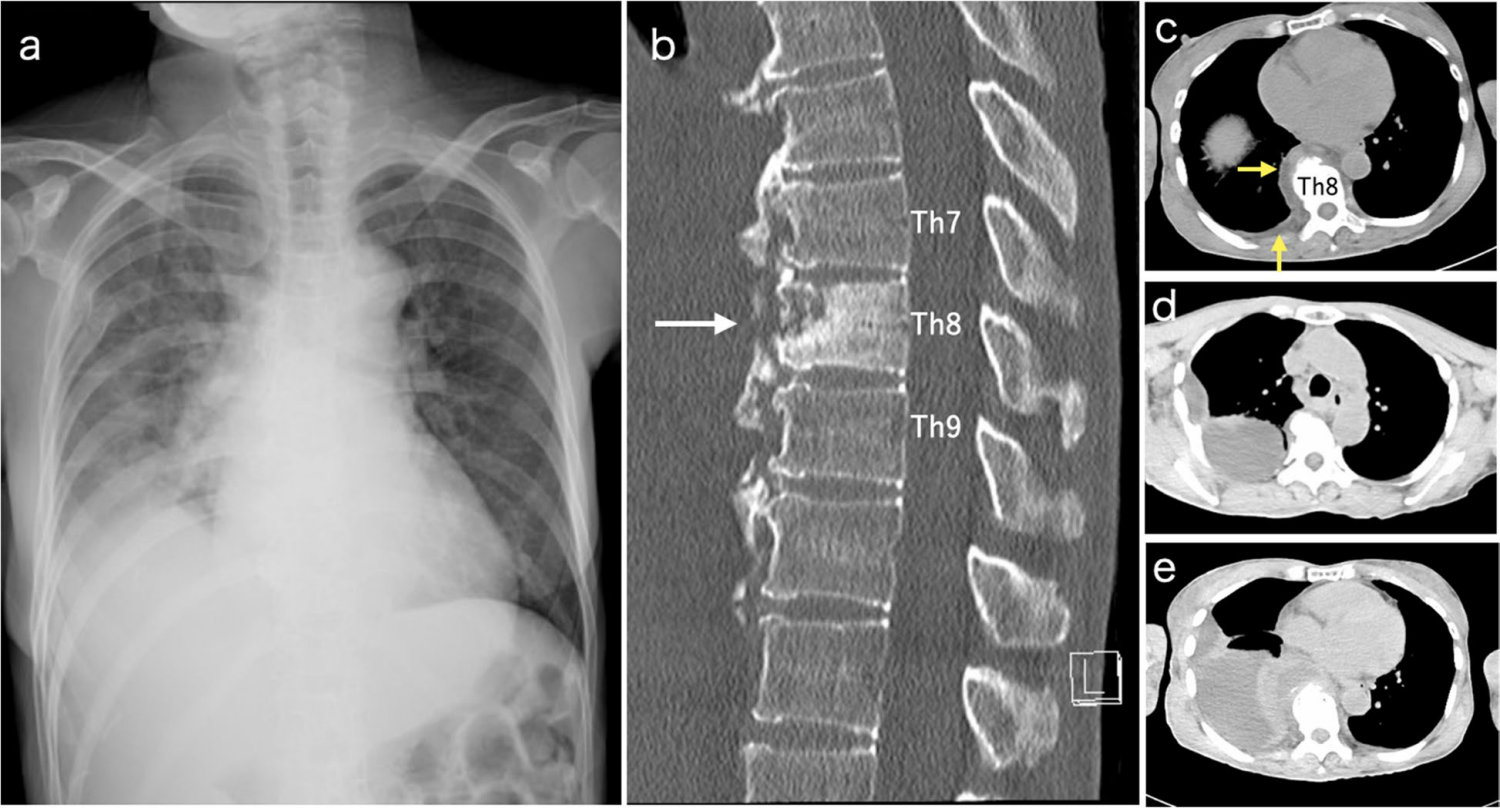
**a** A chest radiograph shows decreased right lower lung field permeability. **b**, **c** Chest computed tomography (CT) presents osteolytic and osteoid changes in the eighth thoracic vertebra (white arrow) (bone window), with surrounding soft tissue thickening and minimal right pleural effusion (yellow arrows) (mediastinal window) on admission. **d**, **e** Chest CT shows a right encapsulated pleural effusion on the eighth hospital day (mediastinal window)

A 60-year-old female with a history of cellulitis, lumbar pyogenic spondylitis, and iliopsoas abscess due to repeated falls related to Parkinson's disease was admitted to our hospital. The patient’s chest radiography revealed decreased permeability in the right lower lung field (Fig. 1a). Laboratory examination revealed an elevated inflammatory response (white blood cells [WBCs], 18,600/μL; neutrophil [Neut] level, 93.8%; and C-reactive protein [CRP], 10.46 mg/dL). Chest computed tomography (CT) revealed a compression fracture of the eighth thoracic vertebra, surrounding soft tissue thickening, and minimal pleural effusion in the right thoracic cavity (Fig. 1b, c). The patient was diagnosed with pyogenic spondylitis associated with a compression fracture of the eighth thoracic vertebra; the patient was admitted to the department of internal medicine, and cefazolin was administered. The pleural effusion was reactive and was not considered a pyothorax at this point.

**Fig. 2:**
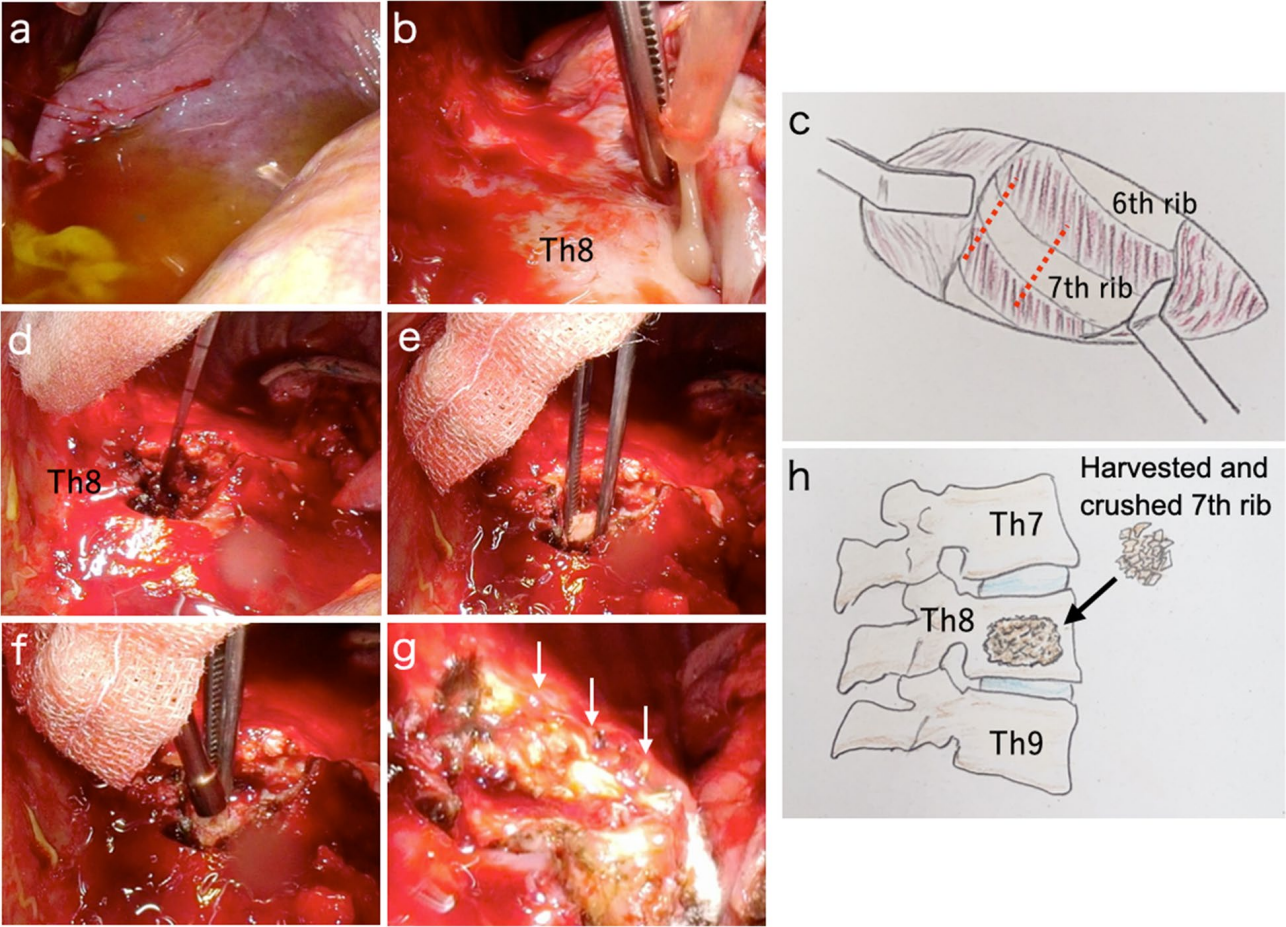
**a** Serous pleural effusion in the thoracic cavity. **b** White purulent effusion when dissecting the strong adhesion between the eighth thoracic vertebra and lung parenchyma. **c** Schema of wound and rib harvesting. A seventh rib was excised 5 cm along the red dotted line. **d**, **e**, **f** Debridement of the eighth thoracic vertebra and an anterior fixation by grafting a harvested rib were performed. Anterior fixation was achieved by only filling the harvested and crushed ribs into the debrided vertebral space. **g** Findings of the eighth thoracic vertebra after anterior fixation (white arrows). **h** Schema of grafting the harvested rib

However, MRSA was identified as the etiologic agent on a blood culture performed on admission; the patient’s antibiotics were changed to vancomycin on the third hospital day. Despite the treatment, there was no improvement in the patient’s inflammatory response (WBC, 13,920/μL; Neut, 88.1%; CRP, 16.42 mg/dL), and CT revealed pleural effusion with encapsulation on the eighth hospital day (Fig. 1d, e). Thoracentesis was performed, and the pleural fluid culture had no bacteria; however, the WBCs increased to 3+. Clinical findings suggested that the right acute pyothorax was caused by pyogenic spondylitis of the eighth thoracic vertebra. The patient was referred to the thoracic surgeon for a discussion on the indication for surgery. No finding suggested spinal canal stenosis, such as paralysis, muscle weakness, or numbness in the lower extremities. However, long-term conservative treatment with antibiotics could cause further antimicrobial resistance, and surgical control of the infected lesion was considered preferable. Curettage of the thoracic cavity abscess and vertebral debridement and anterior fixation were performed simultaneously on the 15th hospital day. The location of the eighth thoracic vertebra was confirmed using X-ray fluoroscopy, and an open thoracotomy was performed through a 15-cm skin incision at the seventh intercostal space, directly above the eighth thoracic vertebra. Serous pleural effusion was present in the thoracic cavity (Fig. 2a), and a white purulent effusion was observed while dissecting the strong adhesion between the eighth thoracic vertebra and lung parenchyma (Fig. 2b). After curettage of the thoracic cavity abscess, 5-cm resection of the seventh rib was performed (Fig. 2c). Crushing of the harvested rib, debridement of the eighth thoracic vertebra, and bone grafting for anterior fixation were performed by orthopedic surgeons with the same wound and view (Fig. 2d–h). Anterior fixation was achieved by filling the crushed rib into the debrided vertebra and pressing them into the space. The thoracic cavity was thoroughly washed with 5 L of saline solution. Total operative time was 168 min, of which 37 min were spent by the orthopedic surgeon. The total volume of aspirated pleural fluid and blood loss was 890 mL and the hemoglobin dropped to 8.7 g/dL (preoperatively 11.0 g/dL); therefore, two units of red cell concentrate were transfused intraoperatively.

As the pleural effusion decreased, the thoracic drain was removed on the fourth postoperative day, and the patient was discharged once on postoperative day 20. In addition, MRSA was also detected in pus collected intraoperatively, and oral vancomycin was continued after discharge.

**Fig. 3:**
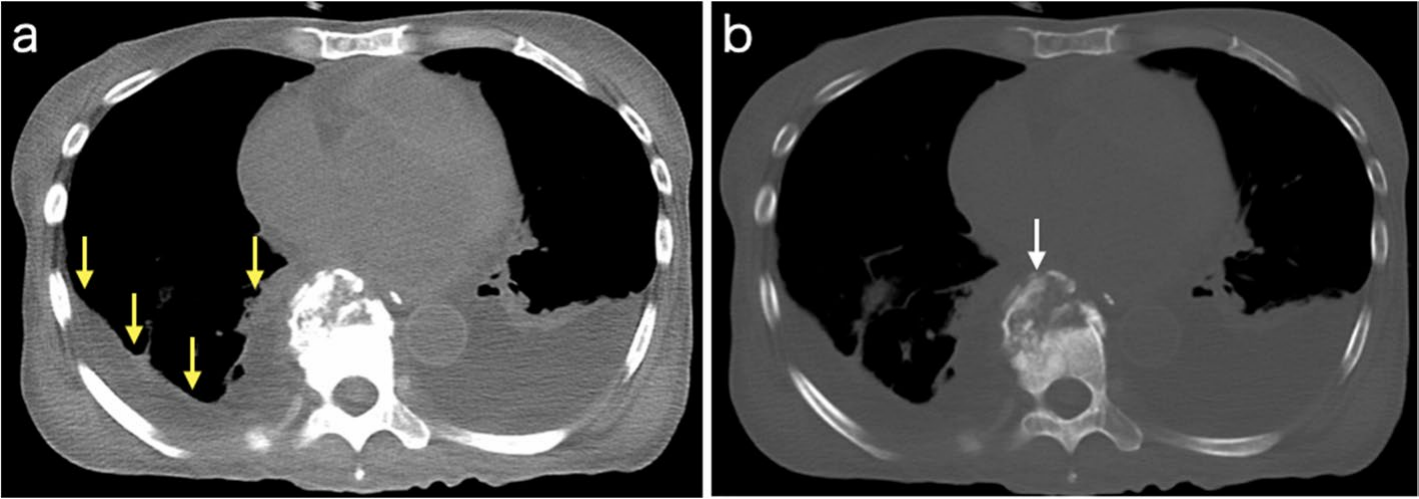
**a** Chest computed tomography (CT) reveals a left pleural effusion (mediastinal window). Although a small amount of right pleural effusion remained (yellow arrows), perivertebral soft tissue thickening has improved 2 months postoperatively. **b** Chest CT shows the incomplete fusion of the grafted bone (white arrow) but there is no evidence of progressive vertebral body destruction or spinal canal stenosis (bone window)

**Fig. 4:**
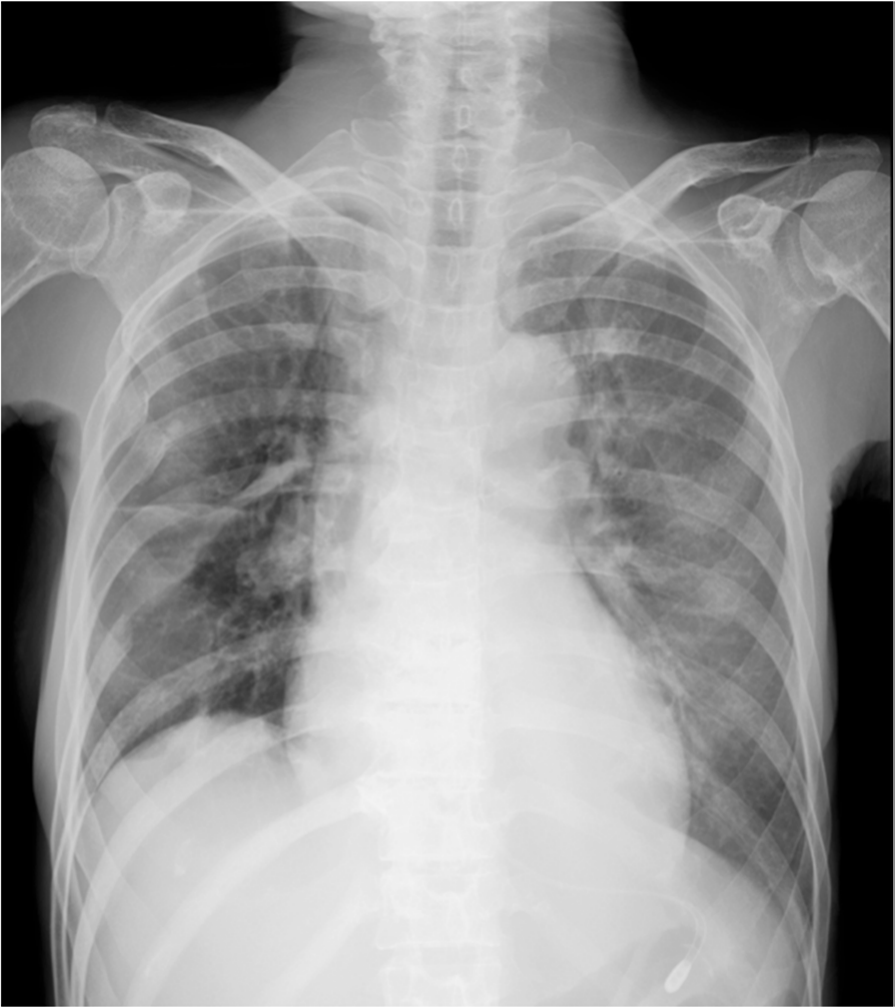
A chest radiograph shows good right lung expansion and permeability improvement. Recurrence of pyothorax was not observed 3 months postoperatively

The right pleural effusion on the chest CT 2 months postoperatively did not worsen (Fig. 3a); and no fever or elevated inflammatory response was observed (WBC, 4710/μL; Neut, 84.5%; CRP, 0.21 mg/dL), the infection was considered controlled, and the oral vancomycin was discontinued. However, the patient had unexplained prolonged hypoglycemia and a poor nutritional status (total protein, 5.1 g/dL; albumin, 2.7 g/dL), which required management via tube feeding. No other findings suggested cardiac or hepatic dysfunction; therefore, the left pleural effusion revealed by the CT was considered transudative based on hypoalbuminemia (Fig. 3a). The fusion of the grafted bone was incomplete (Fig. 3b); however, there was no evidence of progressive vertebral body destruction or spinal canal stenosis. Therefore, the fusion state was considered satisfactory without apparent issues. A chest radiography 3 months postoperatively revealed no recurrence of pyothorax (Fig. 4). However, the patient was transferred to another hospital for long-term rehabilitation due to Parkinson's disease.

## Discussion and conclusions

This case demonstrated that, first, MRSA spondylitis can cause acute pyothorax. Second, simultaneous surgical treatment for spondylitis and pyothorax is effective.

In the present case, thoracic vertebral compression fracture, surrounding soft tissue thickening, abscess formation in the thoracic cavity adjacent to the compression fracture, and detection of the same pathogen (MRSA) in blood and intrathoracic abscess cultures led to the conclusion that MRSA pyogenic spondylitis developed at the compression fracture site of the eighth thoracic vertebra. In contrast, pyothorax developed from the direct spread of infection into the thoracic cavity. *Staphylococcus aureus* [1], *Mycobacterium tuberculosis* [6–8], and *Streptococcus gordonii* [2] have been reported as causative agents of similar conditions. In addition, rare cases due to *Salmonella* [12, 13] and *Mycobacterium abscessus* [11] have been reported. However, there have been no reports of MRSA pyogenic spondylitis (Table 1).

**Table 1:** Summary of patients with acute pyothorax due to thoracic pyogenic spondylitis or intervertebral discitis

Case	References	Age (years)	Sex	Pathogen	Th level	Pyothorax side	Procedures
1	Lang [6]	UNK	UNK	*Mycobacterium tuberculosis*	UNK	UNK	UNK
2	Bloom et al. [1]	49	M	*Staphylococcus aureus*	10	Right	Evacuate paraspinal abscess
3	Bloom et al. [1]	63	M	*Staphylococcus aureus*	7–8	Left	Evacuate necrotic bone
4	Hendrix et al. [9]	57	M	*Aspergillus fumigatus*	1–5	UNK	Thoracic drainage Decompressive laminectomy from Th1-5
5	Sullivan et al. [10]	64	M	*Staphylococcus aureus*	7	Bilateral	Costotransversectomy and vertebral bone biopsy Thoracentesis
6	Shimada et al. [12]	75	F	*Salmonella*	10–11	Bilateral	None
7	Bass et al. [5]	74	F	*Proteus mirabilis*	9–11	Right	Thoracic drainage, Laminectomy from Th9-11
8	Bass et al. [5]	62	M	*Streptococcus agalactiae*	6–7	Right	Thoracic drainage, thoracotomy, and decortication
9	Prasad et al. [7]	67	M	*Mycobacterium tuberculosis*	12	Left	Thoracic drainage
10	Zheng et al. [13]	42	M	*Salmonella*	12	UNK	CT-guided psoas muscle abscess drainage
11	Taniguchi et al. [14]	59	F	*Staphylococcus Aureus*	11–12	Bilateral	Thoracic drainage Anterior fixation with autogenous bone (ilium)
12	Ruzicić et al. [8]	UNK	UNK	*Mycobacterium tuberculosis*	10–11	UNK	Decortication
13	Nakamura et al. [2]	74	M	*Streptococcus gordonii*	12	Right	Thoracoscopic curettage
14	Kadota et al. [11]	63	F	*Mycobacterium abscessus*	UNK	Right	Thoracoscopic curettage
15	Tatara et al. [3]	60	F	*Streptococcus Anginosus Group*	8–9	Bilateral	Curettage through open thoracotomy Discectomy and non-instrumented fixation
16	Bonnesen et al. [4]	77	F	*Fusobacterium nucleatum*	10–11	Left	Thoracic drainage
17	Altunçekiç et al. [15]	17	F	*Brucella*	11–12	Bilateral	Thoracic drainage
18	Current case	60	F	MRSA	8	Right	Curettage through open thoracotomy Thoracic vertebral debridement Anterior fixation with autogenous bone (rib)

*UNK* unknown, *M* male, *F* female, *Th* thoracic, *CT* computed tomography, *MRSA* methicillin-resistant Staphylococcus aureus

Reports of similar cases have revealed varying surgical treatment strategies, including curettage of the thoracic cavity [2, 11], thoracic drainage and long-term antimicrobial therapy [4], right open thoracotomy, discectomy, and vertebral body fixation [3]. Despite the low invasiveness of conservative treatment, prolonged inflammation may occur owing to poor drainage, and prolonged administration of antibiotics may lead to antimicrobial resistance. In some cases, the administration of antibiotics had to be discontinued due to side effects caused by long-term administration [12], or surgery was required due to worsened disease conditions after conservative treatment [2, 5]. MRSA is a resistant bacterium; therefore, developing further resistance could be disadvantageous for disease improvement. In contrast, surgery is invasive and associated with postoperative pain and the risk of new wound infection. However, early curettage of the thoracic cavity and vertebral debridement and anterior fixation may effectively drain the thoracic cavity and vertebral body and reduce the risk of bacterial resistance by shortening the duration of antimicrobial use. Fortunately, changing antibiotics owing to recurrent inflammatory reactions or new bacterial resistance was unnecessary.

In the present case, resecting the rib as the autogenous bone had some disadvantages, such as pulmonary herniation and increased pain; nonetheless, we prioritized the advantages of reduced infection risk and less invasiveness by not creating an extra wound. Tatara et al. performed nonartificial fixation, similar to the present case, but added screw fixation for residual spinal canal stenosis [3]. Implanting an artificial device near an infected lesion may further increase infection risk; however, it is important to understand the disadvantage of autogenous bone fixation, which can also cause pain at the harvest site and poor fusion in older patients [16].

In conclusion, acute pyothorax is rare but may be caused by pyogenic spondylitis, and MRSA is an even rarer causative agent. Simultaneous debridement and anterior fixation of the vertebral body with curettage of the thoracic cavity abscess may be effective in its management strategies.

## Data Availability

Not applicable.

## Abbreviations

MRSA: Methicillin-resistant *Staphylococcus* aureus
WBC: White blood cell
Neut: Neutrophil
CRP: C-reactive protein
